# Emerging Roles of DLK1 in the Stem Cell Niche and Cancer Stemness

**DOI:** 10.1369/00221554211048951

**Published:** 2021-10-04

**Authors:** Elisa Stellaria Grassi, Alexander Pietras

**Affiliations:** Department of Medical Biotechnology and Translational Medicine, University of Milan, Milan, Italy; Division of Translational Cancer Research, Department of Laboratory Medicine, Lund University, Lund, Sweden

**Keywords:** angiogenesis, cancer, DLK1, glioblastoma, stem cell, stemness, tissue differentiation, tumor heterogeneity, tumor immune infiltrate, tumor microenvironment

## Abstract

DLK1 is a maternally imprinted, paternally expressed gene coding for the transmembrane protein Delta-like homologue 1 (DLK1), a non-canonical NOTCH ligand with well-described roles during development, and tumor-supportive functions in several aggressive cancer forms. Here, we review the many functions of DLK1 as a regulator of stem cell pools and tissue differentiation in tissues such as brain, muscle, and liver. Furthermore, we review recent evidence supporting roles for DLK1 in the maintenance of aggressive stem cell characteristics of tumor cells, specifically focusing on central nervous system tumors, neuroblastoma, and hepatocellular carcinoma. We discuss NOTCH -dependent as well as NOTCH-independent functions of DLK1, and focus particularly on the complex pattern of DLK1 expression and cleavage that is finely regulated from a spatial and temporal perspective. Progress in recent years suggest differential functions of extracellular, soluble DLK1 as a paracrine stem cell niche-secreted factor, and has revealed a role for the intracellular domain of DLK1 in cell signaling and tumor stemness. A better understanding of DLK1 regulation and signaling may enable therapeutic targeting of cancer stemness by interfering with DLK1 release and/or intracellular signaling.

## Introduction

Delta-like homologue 1 (DLK1) is a transmembrane protein that belongs to the NOTCH non-canonical ligand family and plays an important role in the regulation of stem cell pools, tissue differentiation during development, cancer differentiation, and cancer stem-like cell (CSCs) maintenance.^1–11^ It is coded by a maternally imprinted, paternally expressed gene localized on chromosome 14 in human and chromosome 12 in mouse.^2,12–14^

**Figure 1.**
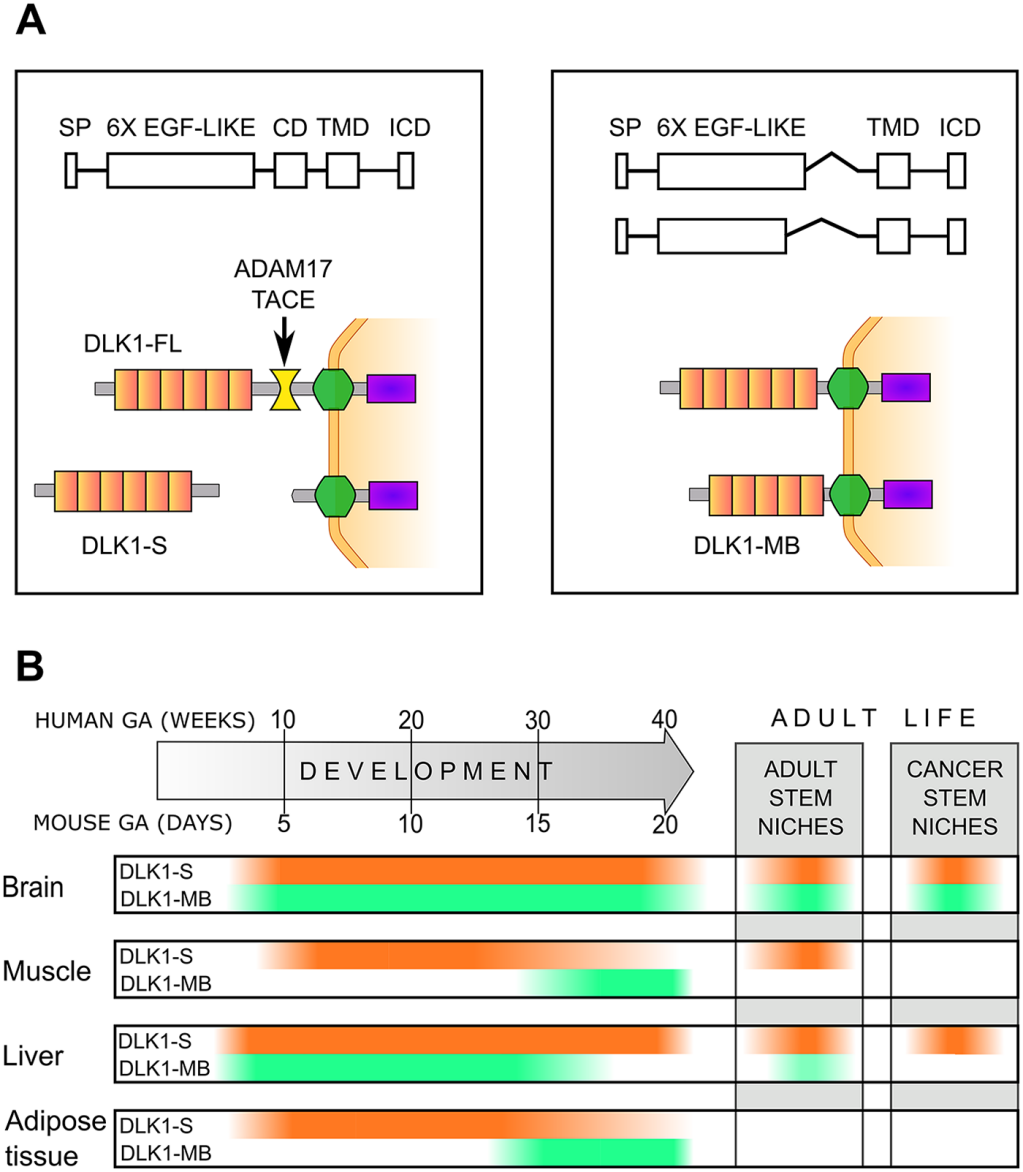
DLK1 isoforms play different roles in tissue development and stem cell pool maintenance. (A) DLK1 is composed of six epidermal growth factor (EGF)–like tandem repeats that constitute the major part of the extracellular domain (ECD), an ADAM17/TACE cleavage domain (CD), a transmembrane domain (TMD), and a short intracellular domain (ICD). Alternative splicing can generate different isoforms that lack the ADAM17/TACE cleavage site and part or all of the sixth EGF-like repeat and are thus membrane-bound (DLK1-MB). The cleavage of full-length DLK1 generates the secreted form of the protein (DLK1-S) that may act as a paracrine factor. (B) Variations in the levels of DLK1-S (orange) and DLK1-MB (green) during development and in stem cell pools and in cancer stem cell niches of adults. The DLK1 timelines represent an integration of the different data available in mouse and humans.

DLK1 exists in different forms. The full-length protein is composed of six epidermal growth factor (EGF)–like tandem repeats that constitute the major part of the extracellular domain (ECD), a transmembrane domain (TMD), and a short intracellular domain (ICD).^15,16^ The presence of an ADAM17/TACE cleavage site in the juxtamembrane region allows the release of a soluble form of DLK1^15^ and of the ICD.^8^ Moreover, alternative splicing can generate different isoforms that lack the ADAM17/TACE cleavage site and part or all of the sixth EGF-like repeat, and are thus membrane-bound^16–20^ (Fig. 1A). Whereas in humans it is present only in one membrane-bound and one full-length cleavable form, in mouse, the pattern is more complex, with four membrane-bound forms and two full-length forms.^19,21^

Irrespective of the isoform, DLK1 mechanisms of action involve both NOTCH-dependent and NOTCH-independent pathways. Although lacking the Delta/Serrate/LAG-2 domain that is necessary for the interaction between NOTCH and its canonical ligands,^2^ DLK1 can directly interact with NOTCH1 through its fifth and sixth EGF-like domains.^22,23^ Anyway, the role of DLK1 in NOTCH pathway modulation is still controversial, as many studies reported contrasting results,^3,23–33^ probably depending on the experimental setting and the DLK1 isoform studied. DLK1 can also interact with itself, through different EGF-like domains than the ones used for the NOTCH interactions, and the degree of DLK1 self-interaction can modulate the ones with NOTCH.^34^ In this scenario, the expression of different DLK1 forms and the relative membrane-bound to soluble ratio can greatly influence the final outcome.^26,35,36^

Apart from the NOTCH pathway, DLK1 can also interact with other partners as, for example, Insulin-like Growth Factor binding protein 1, Fibroblast Growth factor receptor, Fibronectin, and Prohibitins, providing alternative routes for regulating cell differentiation and metabolism.^37^–^39^

The most widely studied function of DLK1 is its role in adipogenesis,^18,24,38,40^ but in recent years, it has emerged that DLK1 plays a fundamental role in cellular differentiation by regulating the maintenance of stem cell pools both in fetal and in adult life.^2,3,41,42^ DLK1 is widely expressed during embryogenesis and tissue differentiation, whereas the levels significantly drop postnatally. In adults, only few tissues retain basal *DLK1* expression, but the levels can rise again after injuries, in various diseases, and in cancer^9,43–53^ (Fig. 1B). The expression of the different DLK1 forms and their role is significantly variable, depending on the developmental stage, on the tissue, and on the cancer type.^1,17,54–56^

## DLK1 and Stemness Modulation in Development and Tissue Regeneration

As many other NOTCH ligands, DLK1 expression is tightly regulated. Different in vivo models with altered *Dlk1* expression reported increased perinatal lethality, growth retardation, and various developmental defects.^46,57–60^ Reduced DLK1 expression leads to premature differentiation of tissues and organs, while DLK1 overexpression delays the differentiation process and increases proliferation of the precursors. Indeed, a tight regulation of DLK1 levels is necessary for a correct timing in the shift between the maintenance of the stem cells pools, the self-replication of the precursors, and the induction of their terminal differentiation in different cellular types.^3,6,41,61,62^ The interplay between the secreted and the membrane-bound DLK1 has been better characterized in the development of brain, muscle, liver, and adipose tissue.

DLK1 plays a fundamental role in the development of various brain structures and in the maintenance of the neural stem cell pool in the adult.^27^ In mouse embryos, Dlk1 expression has been found in the anterobasal nucleus, in the anterior thalamic and septal neuroepitelia, in the ventral pons, and in basal and medial tegmental neuroepitelia.^63^ In adult humans and mice, strong DLK1 expression has been found in the monoaminergic brainstem nuclei in mesencephalon and pons.^63^ Dlk1 is necessary to temporally and spatially regulate the differentiation of neuronal precursors in the different mature neurons type and plays a fundamental role in regulating the appearance of mature mesodiencephalic dopamine neurons.^1,44,64^ Recently, it was demonstrated that biallelic expression of *Dlk1* in the subgranular zone is required for hippocampal stem cell maintenance and neuronal plasticity modulation.^65^ Moreover, mice that lack *Dlk1* expression also have postnatal developmental defects in the subventricular zone and in the olfactory bulb.^3^ In the latter case, the interplay between the different forms of Dlk1 is fundamental for the maintenance of the neuronal stem cells in the subventricular zone germinal niche from the early postnatal period and through the adult life (Fig. 1B). Specifically, specialized niche astrocytes secrete Dlk1 while the neural stem cells express only the membrane-bound isoform. The secreted Dlk1 acts as a niche paracrine factor, and through the membrane-bound form expressed by neural stem cells regulates their number in a Jag1 and Notch1 independent manner. Depletion of Dlk1, either from astrocytes or from neural stem cells, results in differentiation into mature neurons and loss of the stem cell pool.^3^

In the hypothalamus, *DLK1* expression and release of the soluble form increases just after birth, suggesting a role in the postnatal plasticity and maturation of the hypothalamic neurons. Interestingly, the hypothalamus is another active site of neurogenesis in adult life, and here DLK1 acts as a regulator of stem cell pool maintenance and differentiation into mature neurons.^66^

Similar interplays between the membrane-bound and the secreted form of DLK1 expressed on different cell types also regulate the stem cell pool maintenance and the differentiation in other tissues. For example, DLK1 plays also a fundamental role in the regulation of the stem cell differentiation in the muscle, both during development and in regeneration after injuries. Different murine models show that overexpression of *Dlk1* induces an increase in the muscular mass mainly through enhancing the differentiation of the myocyte precursors, the myoblasts, while *Dlk1* knockout causes reduced muscular mass and impaired muscular regeneration after injuries while promoting the survival of self-renewal satellite cells, undifferentiated cells that survive only in specific stem cell niches between the muscle fibers and their basal lamina.^43,67^

The balance between the membrane-bound and the cleaved DLK1 may play a fundamental role, as an in vitro study found that membrane-bound Dlk1 can promote myocyte precursor differentiation and induce hypertrophic myotube formation, while cleaved, soluble Dlk1 has the opposite effect^56^ (Fig. 1B). Considering the direct role of microenvironmental cues in controlling DLK1 cleavage, this further highlights the context dependency of outcomes of DLK1 expression.

After birth, *DLK1* is not expressed in human muscle, but can still be detected in some of the satellite cells. DLK1 levels rise in the presence of different myopathies such as muscular dystrophies, and following intense exercise and injuries.^33,67–71^ These variations in DLK1 expression influence the fate of the satellite cells, toward myogenic differentiation.^67,72^ Interestingly, a lower percentage of DLK1 positive satellites cells have been detected in individuals who were consuming anabolic substances versus the ones who were regularly training, probably as the result of quick and massive induction of differentiation.^42^

A similar mechanism also regulates the human and the murine liver development. In the fetal liver, the hepatoblasts, precursors with high proliferative rate, and bipotential differentiation into both hepatocytes or cholangiocytes express high levels of both membrane-bound and the full-length cleavable secreted form of DLK1.^6,73–75^

In vitro studies on mouse fetal liver precursors revealed that while cells expressing both the membrane-bound and the secreted form of Dlk1 were able to differentiate into hepatocytes, cells expressing only the secreted form were directed toward differentiation into cholangiocytes. The switch in Dlk1 isoforms expression seems to be dependent on fibroblast growth factor stimulation.^74^ Moreover, in human fetal hepatoblasts, the expression of the membrane-bound DLK1 form is also fundamental for the survival of the hematopoietic progenitors and maintenance of liver fetal erythropoiesis, while the expression of secreted DLK1 alone seems to have an adverse effect.^75^

Irrespective of the relative levels of the different isoforms, the sudden drop in total DLK1 levels that happens before birth is fundamental for the bile duct development and full liver maturation^73,76,77^ (Fig. 1B).

In the adult liver, DLK1 re-expression may play a role in the regenerative response to various chronic injuries. In this scenario, there is an increase in the number of the Atypical Ductal Cells that can display a phenotype similar to the bipotential fetal liver precursors and thus play a role in tissue regeneration. In different murine models, a strongly DLK1 positive subpopulation of Atypical Ductal cells has been detected and characterized either as a quiescent liver precursor cells reactivated after injury^78^ or as mature cholangiocytes that transdifferentiated into bipotential cells to replenish the damaged liver with new mature hepatocytes and cholangiocytes.^48,79^

At last, in different in vitro models of glucocorticoids and insulin-induced adipocyte differentiation, a quick temporary increase in the levels of the membrane-bound variant of Dlk1 was observed just after induction of differentiation, while the full-length, cleavable Dlk1 started to decrease just after induction and both became undetectable by the end of the differentiation process.^80–83^ Both the initial overexpression of the membrane-bound DLK1 and the drop in the levels of secreted DLK1 play a role in adipocyte differentiation, and the switch between the two forms of DLK1 is necessary to induce a permissive state for differentiation in response to extracellular stimuli (Fig. 1B). Again, DLK1-mediated effects appear to be dependent on the microenvironmental context, for example, secreted factors present in a stem cell niche. Different reports confirmed that high levels of secreted DLK1 and treatment with the recombinant soluble protein can prevent the differentiation of preadipocytes in mature adipocytes.^15,21,84,85^

Moreover, mice with adipocyte-specific overexpression of full-length Dlk1 have a significant decrease in the amount of adipose tissue, while mice lacking Dlk1 show increased adiposity and fatty liver.^15,21,57^

## DLK1 in Cancer and Cancer Stem Cell Niches

Although DLK1 expression is lost in the majority of tissues after birth, high DLK1 levels are found in common pediatric malignancies, such as neuroblastoma, nephroblastoma, and hepatoblastoma, and in some of the more aggressive and therapy-resistant adult cancers.^8,50,86–91^

In particular, DLK1 overexpression has been associated with increased aggressiveness and poor outcome in glioblastoma (GBM), hepatocellular carcinoma, ovarian, prostate, and lung cancer.^11,47,49,50,90,92,93^

Indeed, more and more evidence suggests that therapy resistance and relapse are mostly due to the survival of cancer cells presenting with the *stemness* phenotype, typically enriched in specialized microenvironmental niches, and that DLK1 can play a role in the regulation and maintenance of this phenotype.

In particular, the role of DLK1 in cancer stem cell niches has been recently well characterized in brain cancer.

**Figure 2.**
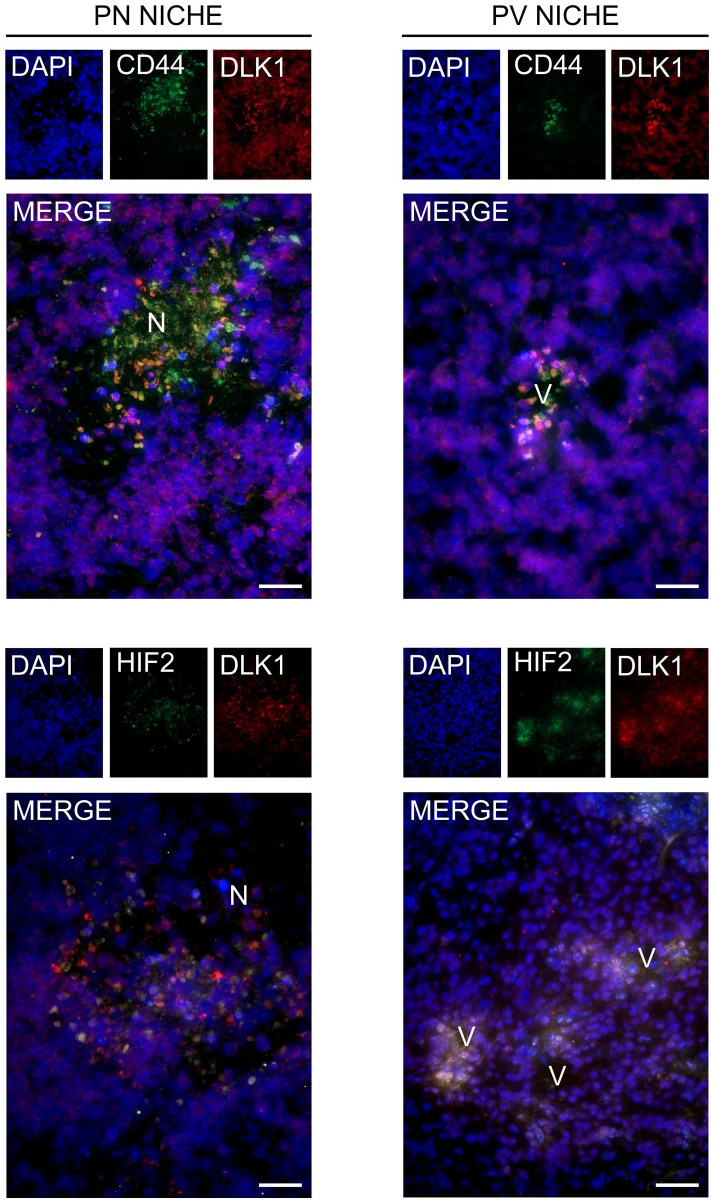
DLK1 expression in glioblastoma (GBM) perivascular and perinecrotic niches. DLK1 expression and colocalization with two markers of the perivascular (PV) and perinecrotic (PN) niches, CD44 and HIF-2alpha, in immunofluorescence performed on cryosections of Nestin/tv-a Ink4a/Arf^−/−^murine GBM induced through neonatal intracranial injection with DF1 cells expressing replication-competent avian sarcoma-leukosis virus long-terminal repeat with splice acceptor (RCAS) encoding human platelet-derived growth factor B (PDGFB) and RCAS-short hairpin p53 (RCAS-shp53). All images were batch processed for background and LUTs optimization. V, vessel; N, necrosis. Scalebars represent 40 µm.

**Figure 3.**
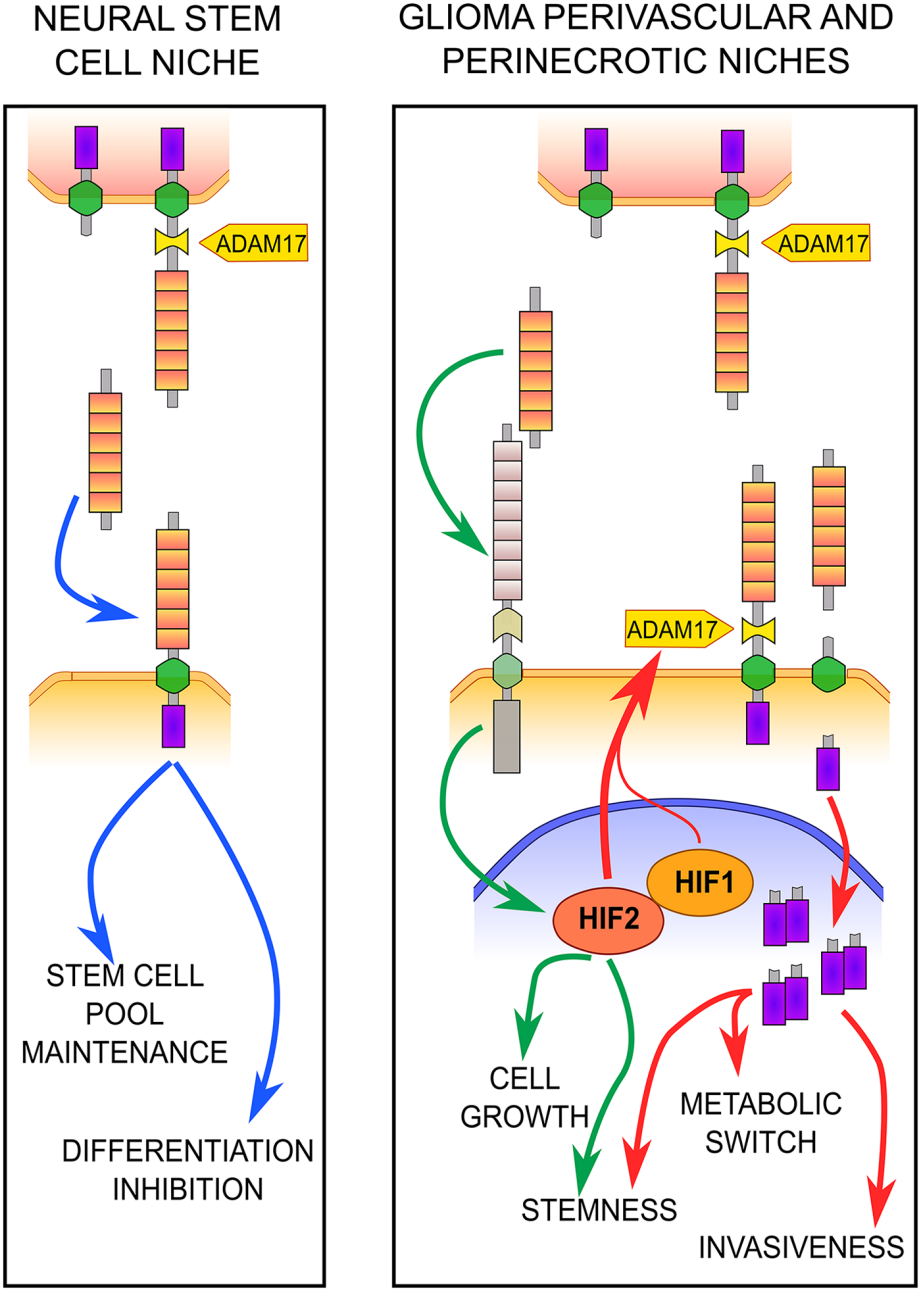
Role of secreted and membrane-bound DLK1 in adult neural stem cell niches and in glioma cancer stem cell niches. In the neural stem cell niches, astrocytes express the full-length, cleavable form of DLK1 (upper cells) while the neural stem cells express the membrane-bound form (bottom cells). The expression of both DLK1 forms in the different cell types is necessary for the inhibition of differentiation and the maintenance of the stem cell pool. In glioma stem cell niches, the reactive astrocyte-secreted DLK1 (upper cells) stimulates cancer cell stemness and growth mainly enhancing HIF-2 activity through a yet unidentified pathway. Moreover, in the cancer cells (bottom), the activation of HIFs induce an ADAM17-dependent cleavage of DLK1 and the release of the intracellular domain (ICD) that then translocates to the nucleus. Here, DLK1 ICD promotes cancer cell stemness and invasiveness mainly through the induction of a metabolic switch that allows a better adaptation to the hypoxic niche environment.

In GBM, the most aggressive form of glioma, a significant increase in DLK1 has been detected in respect to normal brain tissue and DLK1 overexpression-induced migration, loss of anchorage-dependent cell growth, and dysregulation of cell cycle progression in different GBM cell lines.^11,50^ The first study highlighting DLK1 overexpression in GBM also provided a first insight in the possible differential role of secreted versus membrane-bound DLK1 in GBM biology, as it was reported that conditioned media with secreted DLK1 actively stimulated GBM cells growth.^50^ Interestingly, DLK1 is also one of the most expressed genes in tumor-associated astrocytes of high-grade versus low-grade gliomas,^94^ and the astrocyte-secreted DLK1 plays a fundamental role in the maintenance of the neural stem cell pool in the brain subventricular zone niches.^3^ In contrast to normal healthy brain cells, GBM cells have high plasticity, and even bulk tumor cells can acquire a stem-like phenotype if exposed to specific stimuli usually found in determined microenvironmental areas such as the perivascular and perinecrotic niches.^95–99^ These two specific niches are characterized by an abnormal regulation of hypoxia-inducible factors (HIFs) signaling, that is also responsible for induction of DLK1 expression^7,10,100^ (Fig. 2). We recently demonstrated that, similar to what was observed in the subventricular zone neural stem cell niche,^3^ tumor-associated astrocytes secrete high levels of DLK1, especially when they are activated by hypoxia. In turn, the astrocyte-secreted DLK1 acts as a paracrine factor that promotes glioma cell proliferation, stem-like phenotype, and self-renewal abilities, mainly through the stabilization of HIF-2alpha levels^11^ (Fig. 3). On the contrary, GBM cells also express DLK1 on the membrane, but when cultivated in conditions that promote the maintenance of the stem-like phenotype, they present a more complex DLK1 cleavage.^8,11^ In fact, when exposed to hypoxic conditions and high HIF levels, DLK1 undergoes an alternative ADAM17-dependent cleavage with the release of an intracellular fragment that then localizes to the nucleus of the GBM cells. Although the intracellular fragment has not been characterized yet, it is probably constituted by part of the TMD and ICD that are detected as a dimer. Although never described during development and in other physiological conditions, the appearance of the DLK1 intracellular fragment plays a fundamental role in the adaptation of GBM cells to hypoxia. In fact, DLK1 cleavage not only enhances the expression of different stem cell markers but also regulates cell metabolism, allowing the shift in the glucose metabolism that plays a major role in stem-like cancer cells survival under hypoxic condition^8^ (Fig. 3).

Interestingly, in neurobastoma, another tumor in which DLK1 is highly expressed under hypoxic conditions and where it plays a role in the maintenance of a stem-like phenotype of the cancer cells, DLK1 ICD plays a fundamental role.^10^ In this tumor setting, in fact, two highly conserved phosphorylation sites localized in the ICD are necessary for the pro-stemness activities of hypoxia-induced DLK1, as ablation of the phosphorylation sites or deletion of the whole DLK1 ICD results in a significant reduction in the cancer cells’ clonogenic abilities.^10^

A similar role for DLK1 as a stem cell niche factor was also described in another type of cancer. In hepatocellular carcinoma, a distinct DLK1 positive subpopulation with high stemness was recently identified, and characterized by features such as a high capacity to form primary and secondary spheres in serum-free media, the expression of stemness markers, and the ability to form tumors in vivo.^49,101^ Interestingly, DLK1 is also highly expressed in hepatic stem cells and progenitors, and may thus regulate the stemness phenotype of liver cells.^73,76,88,102^ Moreover, the treatment of hepatocellular carcinoma cells with different commonly used anticancer drugs caused an increase in the fraction of the DLK1-positive cells, indicating that these cells may be also responsible for therapy resistance.^101^

In conclusion, DLK1 plays an important role in the regulation of stem cell pools both in tissue differentiation during development and in different neoplasia. In recent years, progress in DLK1 research identified a complex pattern of DLK1 expression and cleavage that is finely regulated from a spatial and temporal perspective and that plays a major role in the differentiation of mature cell precursors both in the fetal and in the adult life. In a similar manner, DLK1 also plays a fundamental role in regulating cancer cell plasticity toward a less differentiated, more stem-like phenotype that may confer increased aggressiveness and therapeutic resistance. Nonetheless, significant differences have been detected between DLK1 expression and subcellular localization between physiological and pathological progress. A better understanding of these differences and of DLK1 role in cancer stemness may open the door for therapeutic targeting approaches, thus providing an alternative tool for fighting highly lethal cancer types.
